# Gender Awareness in Healthcare: Contextualization of an Arabic Version of the Nijmegen Gender Awareness in Medicine Scale (N-GAMS)

**DOI:** 10.3390/healthcare11040629

**Published:** 2023-02-20

**Authors:** Bayan Shamasneh, Maysaa Nemer, Niveen M. E. Abu-Rmeileh

**Affiliations:** Institute of Community and Public Health, Birzeit University, Birzeit P.O. Box 14, West Bank, Palestine

**Keywords:** gender awareness, gender stereotypes, gender in healthcare, Palestine

## Abstract

Gender is one of the important social determinants of health known to be highly associated with health status. Despite the importance of gender awareness, it has not been addressed and researched in the Arab region, including Palestine. This study aimed to contextualize an Arabic version of the Nijmegen Gender Awareness in Medicine Scale (N-GAMS) and to assess the level of gender awareness and its associated factors among primary health care providers. The N-GAMS tool was translated and adapted through a gender expert consultation and a focus group discussion. Then, it was administered online to a sample of primary health care general physicians and nurses of all health care providing actors in Ramallah and al-Bireh Governorate. The reliability of the N-GAMS subscales using Cronbach’s alpha (α) was 0.681 for the gender sensitivity (GS) scale (9 items), 0.658 for the gender role ideology towards co-workers (GRIC) scale (6 items), and α = 0.848 for the gender role ideology towards patients (GRIP) scale (11 items). The results showed that participants had scored near the midpoint of the gender sensitivity subscale (M = 2.84, SD = 0.486). They also expressed moderate gender stereotypes towards patients (M = 3.11, SD = 0.624), where females held lower stereotypical thinking. Participants also expressed low to moderate stereotypes towards co-workers (M = 2.72, SD = 0.660) and females expressed less stereotypical thinking compared to males. Furthermore, the participant’s age had some effect on the outcome, specifically on the GRIP subscale, while gender was associated with both GRIP and GRID subscales. The rest of the social and other variables showed no association with the gender awareness subscales. This study adds to our understanding of gender awareness. Further tests are required to confirm the psychometric qualities of the instrument.

## 1. Introduction

While males and females differ biologically (sex), they also differ in the assigned social responsibilities and roles (gender) [1,2]. Sex is associated with secondary sex characteristics such as sex hormones, sex chromosomes, internal reproductive organs, and external genitalia [3,4]. On the other hand, gender refers to “the culturally defined roles, responsibilities, attributes, and entitlements associated with being (or being seen as) a woman or man in a given setting, along with the power relations between and among women and men” [5]. As a result, gender health differences between women and men exist [6], and gender as a social determinant of health is considered a significant driver of these differences [7,8,9].

Statistical data and research have documented significant gendered differences in health between women and men [10,11,12,13]. These differences include the gender differences in mortality and morbidity rates [11,14,15], reporting and experiencing symptoms of illnesses or risk factors [16,17,18,19], perception of health, the use of preventive measures, the use of drug prescriptions, and referrals to or acceptance of specific surgical treatments [16,19].

Further, as suggested by Heise and colleagues, there are multiple gendered pathways to health. These pathways are gendered differences in exposure, gendered health behaviors, gendered impacts on accessing care, gender-biased health systems, gender-based health research, and institutions and data collection [5].

During the previous two decades, gender awareness has been recognized as a critical factor in the interaction between health care professionals and patients, thus affecting health outcomes for both women and men [17]. The World Health Organization (WHO) defines gender awareness as “understanding that there are socially determined differences between women and men based on learned behavior, which affect their ability to access and control resources” [20]. Further, gender awareness, as defined by the European Institute for Gender Equality, is the “ability to view society from the perspective of gender roles and how this has affected women’s needs in comparison to men’s needs” [21].

Thus, gender awareness conveys that the health care worker holds gender-sensitive attitudes, the knowledge and understanding of the whole meaning and sense of gender in illness and health, and the skills required to apply their visions to the medical practice [22,23]. Gender awareness affects the health care professional’s interactions with their patients [24,25,26,27]. Gender awareness is essential for making genuine connections with patients and contributes to achieving a higher health care quality delivered for both women and men [28,29]. Additionally, health care professionals’ gender awareness can reduce health gender biases [29,30,31], a potential domain that affects gender equality in health care [22,32]. Several studies have discussed that increasing gender awareness among health care professionals may elevate gender equity in health [22,32].

Previous research has operationalized the concept of gender awareness in healthcare into three main sub-components: gender sensitivity, gender-role ideology, and knowledge [29,33,34]. The literature has suggested only two validated scales that offer a theoretically based, multidimensional (minimum of two subcomponents) assessment of gender awareness among health care professionals and medical students [32]. One of these scales is the Nijmegen Gender Awareness in Medicine Scale (N-GAMS), developed by Verdonk and colleagues. They argued that both (social and biological) visions are essential; consequently, they adopted a broader concept of gender awareness, focusing on attitudinal components of gender awareness [29]. The original N-GAMS assesses three dimensions of gender awareness: gender sensitivity, gender-role ideology towards patients, and gender-role ideology towards medical students [29].

The N-GAMS was used in the literature to assess and compare Dutch and Swedish medical students’ gender awareness [35]; evaluate the impact of an intervention program regarding female reproduction, clinical practices of gynecology and obstetrics, and other women’s health-related issues in medical students’ levels of gender awareness [17]; compare differences in general practitioner trainees’ gender awareness following different gender medicine programs [36]; explore the gender awareness of medical students and allied health professions students [37]; assess the level of gender awareness among primary health care physicians and doctors-in-training in Italy [26]; and determine the level of gender awareness among a sample of Swiss medical students and validate the tool in a French-speaking country [21]. These studies highlighted the relevance and applicability of this scale in several contexts, namely, to assess cultural differences in gender awareness and the efficacy of gender training programs focused on increasing gender awareness [32].

Despite the importance of gender awareness, it has not been addressed and researched in the Arab region generally and in Palestine specifically. Additionally, no previous studies have measured gender awareness among health care professionals, and there is no validated tool in Arabic. Therefore, this study aims to contextualize the internationally validated quantitative tool (N-GAMS) that measures gender awareness in health care, through translating and adapting an Arabic version of the N-GAMS among primary health care (PHC) general physicians and nurses in Ramallah and Al-Bireh Governorate, Palestine.

## 2. Materials and Methods

### 2.1. Translation and Adaptation of the Tool

#### 2.1.1. Description of the N-GAMS Tool

The N-GAMS questionnaire assesses the gender awareness levels among health care workers. This scale is divided into three subscales: (1) gender sensitivity (GS): the degree to which health care professionals/medical students are sensitive and sympathetic to the impact of gender in medical practice (14 items); (2) gender-role ideology towards patients (GRIP): health care providers’ stereotypical views towards male and female patients (11 items); and (3) gender-role ideology towards co-workers (GRIC): health care providers’ stereotypical views towards male and female co-workers (7 items) [29]. Answers are assessed on a 5-point Likert scale ranging from 1 (“totally disagree”) to 5 (“totally agree”).

#### 2.1.2. Forward and Backward Translation of the N-GAMS

The forward and backward technique was used to produce (adapt) a linguistic equivalent Arabic version of the scale. First, two bilingual independent translators did the forward translation (from English to Arabic). One translator was aware of the concepts the questionnaire intended to measure, and the other was a naïve translator (unaware of the measured concept of the questionnaire). Then, the translation discrepancies were discussed and resolved between the two translators. The next stage (backward translation) was done by two independent translators (mother tongue is English) who were unaware of the questionnaire concept. Then, both backward translation versions were compared for discrepancies between each other and the original English version. Afterwards, both (Arabic and English) versions were sent to a gender expert to confirm that the translated version was equivalent to the original version. Finally, recommendations from the gender expert and any discrepancies were discussed and applied by the research team.

### 2.2. Focus Group Discussion

One focus group discussion was held, which included a total of 6 participants. Their occupations varied, including nurses, physicians, and pharmacists. The focus group was done to confirm that the Arabic version of the questionnaire is understandable and reflects the same ideas as the original version.

### 2.3. Pilot Study

The questionnaire was piloted at two stages to increase its face validity. The first pilot stage was done on health center staff consisting of one male physician, two female nurses, and one male nurse. Each participant was interviewed face-to-face. Participants evaluated the clarity of the instructions and items of the questionnaire (clear or unclear). When the item or the instruction was evaluated as unclear, the participant was asked to suggest how to improve the clarity. The results from this stage were used to modify the pre-final version of the questionnaire. The pre-final version was used for the final stage (second pilot), which included piloting the questionnaire on one female nurse and one male physician.

### 2.4. Tool Validation

#### 2.4.1. Study Design

We utilized a descriptive (cross-sectional) study design to test the psychometric properties (reliability and validity) of the Arabic version of the N-GAMS, which was translated in the first step of the research. The study was conducted in the period from June to August 2020.

#### 2.4.2. Study Population and Sample

The study took place in Ramallah and Al-Bireh Governorate, in the center of the West Bank, Palestine. The target population (eligible participants) included the PHC professionals (nurses and general physicians) in Ramallah and al-Bireh Governorate. The selection of the sample from the target population was based on three primary strata: (1) type of provider (Ministry of Health (MoH), Non-Government Organizations (NGOs), and the United Nations Relief and Works Agency (UNRWA)), (2) type of health care worker (physicians, nurses), and (3) gender (men, women), proportionally taking into consideration the total target population. The estimated sample size was 150 health care providers.

#### 2.4.3. Data Collection

Before starting the data collection, we obtained permission from the Ramallah and Al-Bireh Health Directorate to invite the PHC general physicians and nurses to participate in the study. Initially, the study was planned to be conducted through field visits to the PHC centers and face-to-face interviews with the general physicians and nurses to collect the data. However, due to the COVID-19 pandemic, this could not be achieved. Thus, we switched to an online alternative to collect the data. First, the survey was created on Google forms. Then, it was piloted on four participants to test the online version of the survey.

The participants were recruited by sending the online survey link to their WhatsApp groups through coordination with administrators of the groups. Two groups were managed by Ramallah and Al-Bireh Health Directorate (one for each profession), and the third group was for UNRWA’s physicians and nurses. We reached out to these groups by contacting members, who sent the link to the rest of the group. Invited participants were sent two to three reminders over three months.

### 2.5. Statistical Analysis

Before starting the analysis, the response set for each individual was examined. We looked for participants who had the same response for all statements on the three subscales and excluded them from the analysis, because it may signal a lack of interest or understanding of the questions.

Descriptive statistics were used to present the participants’ characteristics. The continuous variable (age) is presented as Mean (M) ± Standard Deviation (SD). The categorical variables (gender, social status, place of residence, type of locality, and place of study) are presented as percentages (%). The number of units (n) values is stated for all the variables.

The descriptive statistics of the N-GAMS items included mean ± standard deviation (M ± SD) for each item in the scale and the scored minimum and maximum values. Further, the direction of the negatively worded gender sensitivity items was reversed (all items except GS-1, GS-2, and GS-13, e.g., “physicians should only address biological differences between men and women”; reverse coded). Thus, higher scores reflect higher sensitivity levels. Then the mean scores of each subscale (GS, GRIP, and GRIC) were determined by computing the average values of each subscale’s individual items. Normality testing of the (GS, GRIC, GRIP) computed variables, and the continuous variable (age) showed normal distribution; this was proven by utilizing the Shapiro–Wilk normality test and Q–Q graphs, in addition to the close value of the mean and the terminated mean supporting the normality of the variables.

The internal consistency reliability of the subscales was assessed by measurement of Cronbach’s alpha coefficient (α). For the validity assessment, Bartlett’s test of sphericity and the Kaiser–Meyer–Olkin (KMO)—a measure of sampling adequacy—were used to assess the ability to perform Confirmatory factor analysis (CFA). To support the validity, we tested the following hypotheses: there is a positive correlation between the gender role ideology towards co-workers (doctors/nurses) and the gender role ideology towards patients (hypothesis 1), and an inverse association with gender sensitivity (hypothesis 2).

An Independent sample *t*-test was used to test the association between gender awareness measured by N-GAMS subscales (GS, GRIP, GRIC) (dependent variables) and each independent categorical variable (two-category: sex). Moreover, one-way variance analysis (ANOVA) was used to assess the relationship between the dependent variables and the independent categorical variables (three or more categories), including marital status, place of residence, locality, highest education level, and study country. Further, Pearson correlations assessed the statistical significance between the dependent and continuous independent variables (age) and evaluated the relationship between the N-GAMS subscales (GS, GRIP, GRIC). Multiple regression was conducted to assess further the relationship between the dependent and the independent variables based on the Bivariate analysis.

All statistical analyses were conducted using the Statistical Package for Social Sciences (SPSS) version 20.0. A significance level of *p* < 0.05 and a 95% confidence interval (CI) were adopted.

### 2.6. Ethical Considerations

Ethics approval was obtained from the Institute of Community and Public Health’s Research Ethics Committee at Birzeit University, Reference number: 2020 (2-1). All participants provided written informed consent. All methods were performed in accordance with the relevant guidelines and regulations.

## 3. Results

### 3.1. Characteristics of the Participants

The total study sample consisted of 120 PHC nurses and physicians (females n = 105 (87.5%), males n = 15 (12.5%)), (age range: 23–60, Mean = 41.8; SD = 8.61). Nurses made up 90% of the total sample (n = 108); the majority were females (97.2%), (age range 23–58 years, Mean = 44.1; SD = 4.46). Physicians formed only 10% of the total sample, and they were all men, (age range: 27–60 years, Mean = 34.67; SD = 9.33). Table 1 summarizes the characteristics of the study participants for the nurses and physicians separately and combined.

### 3.2. Descriptive Analysis of the N-GAMS Items

Table 2 shows the mean for each item on the scale, standard deviation, and minimum and maximum values. The GS scale item’s mean values ranged from 2.41 to 3.57, the GRIP item’s mean ranged from 2.25 to 3.60, and the GRIC item’s mean values ranged between 2.03 and 3.25. Moreover, answers covered the range (min = 1, max = 5) for most of the items on the three subscales.

Participants, on average, showed low to moderate levels of gender sensitivity (M = 2.84, SD = 0.486). On the other hand, for the gender role ideology toward patients, participants, on average, revealed moderate gender stereotypes towards patients (M = 3.11, SD = 0.624). Lastly, participants, on average, had low to moderate adherence to gender stereotypes (gender role ideologies towards co-workers (nurses/doctors)) (M = 2.72, SD = 0.660).

### 3.3. Validity Analysis

The literature established a strong positive relationship between factor analysis (FA)—a statistical data reduction method—and construct validity [38]. Scholars use FA to explore or confirm the associations between the tool items and to determine the dimensionality (number of dimensions) among the items [39]. FA is divided into two main types; Exploratory factor analysis (EFA) and Confirmatory factor analysis (CFA). EFA is used in the initial phases of the research to explore relationships underlying a set of data, while CFA is used in the advanced research phases to confirm an established theoretical model.

However, to run an FA test on a data set, two main issues must be considered, the strength of correlations between the items and the sample size. In our study, the sample size, according to [22], should be at least five to ten times the number of items on the scale. In our case, we have a total of 26 items; thus, our sample size should at least range from 130 to 260. Regarding the strength of correlations between the items, it is recommended to have a coefficient for the correlation matrix of 0.3 and higher. Further, Bartlett’s test of sphericity [40], and the Kaiser–Meyer–Olkin (KMO) measure of sampling adequacy [41], are two statistical tests used to determine the data’s adequacy (factorability) for the factor analysis [42]. Bartlett’s test must be significant (*p* < 0.05) for the FA to be considered suitable. The KMO index varies from 0 to 1 [42]. In our study, even though the Bartlett test result was significant (*p* = 0.000), the KMO value was 0.673, which is a mediocre value; thus, factor analysis was not possible in this case [42].

Although the construct validity of the instrument (N-GAMS), could not be confirmed through FA, the following hypotheses were assumed and studied to support the construct validity. In compliance with the previous studies [29], we expected a positive correlation between the gender role ideology towards co-workers (doctors/nurses) and the gender role ideology towards patients (hypothesis 1), and an inverse association with gender sensitivity (hypothesis 2). This implies that agreeing attitudes on the effect of gender in health care practices are associated with lower levels of gender stereotypes. Thus, this reinforces the three-domain construct of the N-GAMS.

As shown in Table 3 below, GRIP and GRIC showed a significant strong/positive correlation (r = 0.680, *p* < 0.001), which backs up the presence of a common ground for gender found between GS with GRIP (r = 0.680, *p* < 0.05) and GS with GRIC (r = 0.680, *p* < 0.05). Thus, findings partially support the hypothesis that the components contribute uniquely to the construct of gender awareness.

### 3.4. Reliability Analysis

Cronbach’s alpha (α) was 0.681 for the GS subscale (9 items), 0.658 for the GRIC subscale (6 items), and (0.848) for the GRIP subscale (11 items). Items with an item total correlation lower than 0.2 were removed as shown in Table 2. The gender sensitivity subscale required the most modifications; a total of five items were excluded, as shown in Table 2, due to low item total correlation (items with less than 0.2 item total correlation were excluded) and based on SPSS recommendation related to Cronbach’s alpha value. The GRIC subscale had only one item exclusion, while the GRIP subscale included all the original items. The mean inter-item correlation for the GS scale was 0.21, while it was 0.33 for the GRID scale, and 0.25 for the GRIP scale, reflecting an acceptable inter-item correlation.

Excluding the “weak” items improved Cronbach’s alpha (α) from 0.611 to 0.681 for the GS scale, and 0.642 to 0.651 GRIP scale. The mean inter-item correlation for the GS scale improved to 0.21 from 0.1 and 0.25 from 0.2 for the GRIP scale. Since it is the first time using the Arabic version of the N-GAMS, CFA should still be carried out to test the tool’s structure after deleting the items.

### 3.5. Factors Associated with Gender Awareness among PHC Providers

The independent sample t-test showed significant differences in the mean GRIP and GRIC scores for males and females. GRIP scores in the female group (M = 3.06 ± SD = 0.599) were significantly lower than the male group (M = 3.55 ± SD = 0.645) (t (117) = 2.967, *p* = 0.004, two-tailed) with a mean difference of 0.495 (95% CI, 0.165 to 0.83). GRIC scores in the female group (M = 2.81 ± SD = 0.49) were significantly lower than the male group (M=3.01 ± SD = 0.4340) (t (117) = 5.96, *p* = 0.000, two-tailed) with a mean difference of 0.95620 (95% CI, 0.52 to 1.39). Yet, there were no statistically significant differences between mean GS scores between males and females. When compared according to participants’ age, using the Pearson correlation, a negative significant association between the GRIP subscale and age (r = −0.288, *p* < 0.001) was found. However, GS and GRIC showed no significant association with age where r = −170, *p* >0.05 and r = −0.011, *p* > 0.05, respectively. There were no statistically significant differences in the mean score of the gender awareness subscales with the other variables (marital status, place of residence, locality, highest education level, and study country).

## 4. Discussion

This study aimed to contextualize and adapt an Arabic version of the Nijmegen Gender Awareness in Medicine Scale (N-GAMS) [29], among primary health care (PHC) general physicians and nurses in Ramallah and Al-Bireh Governorate, Palestine.

### 4.1. Validity and Reliability of the Arabic N-GAMS

The three-domain construct of the N-GAMS is partially supported by confirming hypothesis 1 (there is a positive correlation between the gender role ideology toward co-workers (doctors/nurses) and gender role ideology toward patients), the primary and more common test used to assess and investigate the structural construct validity is confirmatory factor analysis (CFA). Research highly suggests using CFA as an appropriate technique to validate instruments/scales by confirming the instrument’s construct [32].

In this respect, factor analysis has specific requirements to be met to have more reliable outcomes. First, it is stressed that an adequate sample should be present. Generally larger sample sizes are better, results based on small samples are not reliable [22]. The required sample sizes are dependent on several elements, such as the number of factors and items per factor, pattern coefficients, missing values, associations between factors, etc. [39]. Generally, the sample size, according to [22], should be at least five to ten times the number of items on the scale. In our case, we have a total of 26 items; thus, our sample size should at least range from 130 to 260. However, in cases where it is impossible to have a large sample size to meet the requirements of FA, scholars should discuss the validity of evidence by relying on the internal structure of other studies [39]. This was partially supported in this study.

Reliability and internal consistency reflect the degree of correlation between the scale items (if the items measure the same construct). Cronbach’s alpha coefficient is one of the most common indictors of internal consistency [43]. Internal consistency of each subscale with Cronbach’s alpha (α) was 0.681 for the GS scale (9 items), 0.658 for the GRIC scale (6 items), and α = 0.848 for the GRIP scale (11 items). However, alpha (α) has no ideal values. Some researchers consider values of 0.7 and higher as ideal. Furthermore, Cronbach’s alpha value is dependent on the number of items in each subscale [43]. For short scales (less than ten items per subscale/domain), Cronbach’s alpha usually will have a lower value impacting the internal consistency. In this scenario, reporting the items’ mean inter-item correlation might be more fitting. According to Briggs and Cheek (1986), 0.2–0.4 is an adequate range for inter-item correlation [43].

Items with an item total correlation lower than 0.2 were removed. Thus, the mean inter-item correlation for the GS scale was 0.21, while it was 0.33 for the GRID scale, and 0.25 for the GRIP scale, reflecting an acceptable inter-item correlation. Since it is the first time using the Arabic version of the N-GAMS, CFA should be still be carried out to test the structure of the tool after the deletion of the items.

Regarding the excluded items, the gender sensitivity subscale required the most modifications, a total of five items were excluded due to low item total correlation (items with less than 0.2 item total correlation were deleted) and based on SPSS recommendation for the Cronbach’s alpha value. The GRIC subscale had only one item exclusion, while the GRIP subscale included all the original items. The exclusion of a high number of items from the subscale was observed in the previous research [32,35]. This could be attributed to the extraction methods used in the N-GAMS construction study [29], where they used principal component analysis, which is a less appropriate method for scale construction [29,32].

### 4.2. The N-GAMS Scale Scores

Measuring gender awareness and the associated stereotypes and attitudes by using a quantitative scale have advantages and disadvantages. Regarding the advantages, employing such a tool will provide the capacity to conduct research and evaluations on the gender awareness topic, while including more individuals from the target population simultaneously, and is cost and time-efficient [35].

On the other hand, since N-GAMS only measures the attitudinal component of gender awareness, a comprehensive understanding of the health professionals’ gender awareness is required. Future studies should assess health care workers’ knowledge on how sex and gender may influence an individual’s health and health care, along with the skills required to incorporate such knowledge in clinical practice [32]. Hence, further qualitative research is needed to provide a more in-depth analysis of health care workers’ underlying logic social discourses [35].

#### 4.2.1. Gender Sensitivity Score

Participants held neutral opinions on the GS subscale statements. In this respect, even though health care workers observe daily disparities between males and females in everyday actions and tasks, their views, expectations, assumptions, and values are also influenced by societal conditions and their behavior [35]. This contradiction might have affected their final scores. However, more qualitative research needs to be done to fully understand the result.

#### 4.2.2. Gender Role Ideologies Scores

GS subscale statements reflect the significance of gender disparities in biology and communication related to clinical practice. However, the GRIP and GRIC subscales statements are explicit and evaluative regarding male and female physicians and doctors; for instance, statements expressed that one gender for patients or physicians is characterized as “too much”, “less”, or “better” than the opposing gender. Therefore, when the health care workers agree with statements, it reflects believing and accepting in gender differences and the hierarchy of these differences. One gender’s characteristics are considered better and more favorable than those of the opposite gender [35]. The statements correspond to societal gender stereotypes and generalizations. Males are labeled as more effective, skilled, and trustworthy than females, who are portrayed as more emotional, concerned, and require attention and time to communicate [35]. Moreover, the health care workers expressed lower/less evident gender stereotypes towards co-workers compared to the stereotypes held towards patients. It has been documented that an individual’s self-reported traits are generally less gender-stereotypical than their assessments of a “typical person’s” [35]. This indicates the presence of in-group favoritism bias, which benefits self-positive social identity [32].

### 4.3. Relationship between the N-GAMS Subscales (GS, GRIP, and GRIC)

Consistent with previous studies [29,32], there is no significant association between gender sensitivity and gender role ideology domains. This suggests that primary health care workers could sympathize with female and male patients’ particular needs, while still agreeing with unfavorable gender stereotypes [29]. Further, this also indicates that the two domains of the gender awareness concept are distinct sub-dimensions of gender awareness that will need to be addressed separately in future interventions [32].

### 4.4. Relationship between Gender Awareness and Age and Gender Variables

Age and gender were the only variables that were significantly associated with some subscales of the N-GAMS. The other background variables could not statistically help to understand the gender awareness levels.

#### 4.4.1. Gender

Gender sensitivity was not significantly different between male and female participants, which was found in other studies [29,37]. On the other hand, females had significantly lower stereotypes towards both patients and co-workers.

Other studies that used the N-GAMS also reported females having/scoring lower with regard to stereotypes than males toward patients and co-workers including Swiss medical students [21], Sweden and Dutch medical students [35], Portuguese medical students [32], medical students and allied health professions [37], and a sample of Italian primary care physicians and doctors-in-training [26].

We should note here that we are aware of comparing our results with studies that used the N-GAMS tool but with different target populations, but we had to due to the limited studies done on similar groups to ours. Males and females differ in terms of the outspokenness of gender role ideologies [2]. In our results, females clearly expressed their disagreement with gender stereotypes counter to males who had more neutral answers. This could be attributed to the fact that these stereotypes are usually related to women’s position and their need to have the appropriate health care [21]. Likewise, men’s lack of interest and curiosity will result in a lack of motivation to address their gendered values, beliefs, and attitudes, thus making them more susceptible to stereotypes and assumptions associated with women’s and men’s desires, needs, actions, and behaviors [35]. Furthermore, males may be more welcoming to accept gender stereotypes because it tends to be more positive about males, which applies to GRIP subscale statements [35].

#### 4.4.2. Age

Older health care workers had a better gender awareness, specifically on the gender role ideologies stereotypes domain, which was also reported in previous studies [32]. Older generally means a longer complex and extensive clinical experience, and this exposure might lead to expressing positive attitudes [32]. Further studies are required on this topic.

### 4.5. Sample

The nurse’s male: female ratio in our sample (97.2% females, 2.8% males) was consistent with the ratio of the target population (96% females, 4% males), based on unpublished data provided by the MoH. By contrast, the physician’s male: female ratio did not reflect the target population ratio; no female PHC physicians have participated in our study, compared to 41% female and 59% male physicians found in the target population.

On the other hand, participants reflected the distribution of physicians and nurses in PHC centers in Ramallah and al-Bireh in terms of the type of the center. A total of 79.2% of the total participants were from MoH centers, 13.3% were from UNRWA, 6.7% were from NGOs and the MoH, and finally, 0.8% were from NGOs. Thus, we could not reach our physician’s target sample size, reflecting the low response rate (12%) from the physicians, especially the females. Research has shown that it might be challenging to conduct surveys of health care professionals. Low response rates are prevalent among health care practitioners in general and physicians in particular [44]. This pattern was also observed in the Palestinian context; for example, Elsous and colleagues surveyed physicians and nurses in two of the major hospitals in the Gaza Strip, where they had a high response rate from the nurses (75.6%) versus a lower (24.4%) response rate for the physicians. The response rate varied drastically between male and female physicians; 96% were males, and only 4% were females. Our study was no exception, physicians had a very low response rate, reaching zero among female physicians, while nurses had a very high response rate [45]. Therefore, we attempted to stimulate their response by implanting different techniques. Initially, the invitation was sent through official MoH representatives and their official channels (WhatsApp groups). The research found more responses when official parties were involved. Further, mixed mode follow-ups were suggested [46]. Thus, reminders were sent through the official channels, telephone follow-ups were done twice, and the link was resent to the preferred platform, personal WhatsApp or e-mail. However, during the phone calls, we captured two reactions from the physicians; some of them were welcoming and willing to participate in the study and others said that they would do it but did not show any positive attitude.

Some possible factors could explain why physicians had a low response rate. Possibly, participants did not find interest in our research topic or found it irrelevant and insignificant. Additionally, it is likely that physicians had a busier schedule and thus had less free time to fill out the survey. Therefore, we addressed this issue by keeping the link accessible for a long period with easy access (available on personal cell phones). Ultimately, we could not eliminate the nonresponse bias, this study lacked the participation of physicians, especially females, which impacted the generalizability of the result and its applicability.

### 4.6. Strength and Limitations

This study attempted to contextualize an Arabic version of an international tool (N-GAMS) measuring the gender awareness concept, which to the best of our knowledge, will be the first Arabic version of the tool. We would like to acknowledge some limitations for this study. First, the results and its applicability were impacted by the small sample size and nonresponse bias. Physicians had a very low response rate of 12% and there was no response from female physicians. Further, psychometric qualities of the Arabic N-GAMS need further testing on larger sample size, as we could not meet the requirements for the CFA. In addition, the results of this study might be affected by the social desirability bias, as the topic is sensitive and the responses were based on self-assessment. Finally, even though this instrument focused on the attitudinal components of gender awareness, qualitative research is needed to understand the other aspects of gender awareness.

## 5. Conclusions

Gender awareness among primary health care physicians and nurses has not been studied extensively globally and in Palestine. This study attempted to contextualize an international tool (N-GAMS) measuring the gender awareness concept in the Palestinian context. However, a larger sample size (a more significant number of both females and males) is required to confirm the psychometric qualities of the instrument along with qualitative research to understand the other aspects of gender awareness.

## Figures and Tables

**Table 1:** General Characteristics of the participants.

Variable/Characteristic	Total		Nurses		Doctors	
Age in years (n = 112) (mean ± standard deviation)	41.8 ± 8.16		42.7 ± 7.68		31.0 ± 9.33	
	n	%	n	%	n	%
Sex (n = 120)						
Male	15	12.5	3	2.8	12	100
Female	105	87.5	105	97.2	0	0
Marital Status (n = 118)						
Single	7	5.9	3	2.8	4	33.3
Married	107	90.7	99	91.7	8	66.7
Divorced	2	1.7	2	1.9	0	0
Widowed	2	1.7	2	1.9	0	0
Place of residence (Governorate) (n = 117)						
Ramallah and Al-Bireh	112	95.2	103	97.2	9	81.8
Jerusalem	3	2.6	2	1.9	1	9.1
Nablus	2	1.7	9.1	0.9	1	9.1
Locality (n = 120)						
Village	81	67.5	76	70.4	5	41.7
City	39	32.5	32	29.6	7	58.3
Highest Education Level (n = 117)						
Diploma	44	37.6	33	31.4	0	0
High Diploma	9	7.7	8	7.6	0	0
Bachelor’s	56	47.9	56	53.3	11	91.7
Master’s	8	6.8	8	7.6	1	8.3
Study Country (n = 110)						
Inside Palestine	88	80	87	84.5	1	14.3
Outside Palestine (Arab Country)	17	15.5	14	13.6	3	42.9
Outside Palestine (non-Arab Country)	5	4.5	2	1.9	3	42.9

**Table 2:** Description of the N-GAMS scale.

Item		M	SD	min	max
GS		2.84	0.486	2.41	3.57
GS1 *	addressing differences between men and women creates inequity in healthcare	2.73	1.071	1	5
GS2 *	physicians’ knowledge of gender differences in illness and health increases the quality of care	3.45	1.095	1	5
GS3_R *	physicians should only address biological differences between men and women	3.31	1.095	1	5
GS4_R	in non-sex-specific health disorders the sex/gender of the patient is irrelevant	3.47	0.99	1	5
GS5_R *	a physician should confine as much as possible to biomedical aspects of health complaints of men and women	3.57	0.935	1	5
GS6_R	physicians do not need to know what happens in the lives of men and women to be able to deliver medical care	3.54	0.937	2	5
GS7_R	differences between male and female physicians are too small to be relevant	3.13	1.005	1	5
GS8_R	especially because men and women are different, physicians should treat everybody the same	2.41	1.003	1	5
GS9_R *	physicians who address gender differences are not dealing with the important issues	3.38	0.813	2	5
GS10_R	in communicating with patients it does not matter to a physician whether the patients are men or women	2.61	1.201	1	5
GS11_R	in communicating with patients it does not matter whether the physician is a man or a woman	3.22	1.114	1	5
GS12_R	differences between male and female patients are so small that physicians can hardly take them into account	3.23	0.961	1	5
GS13	for effective treatment, physicians should address gender differences in etiology and consequences of disease	3.49	0.947	1	5
GS14_R	it is not necessary to consider gender differences in presentation of complaints *	3.27	0.972	1	5
GRIP		3.11	0.624	2.25	3.60
GRIP1	male patients better understand physicians’ measures than female patients	2.25	0.846	1	4
GRIP2	female patients compared to male patients have unreasonable expectations of physicians	2.57	0.953	1	5
GRIP3	women more frequently than men want to discuss problems with physicians that do not belong in the consultation room	3.17	1.011	1	5
GRIP4	women expect too much emotional support from physicians	3.26	1.004	1	5
GRIP5	male patients are less demanding than female patients	3.00	1.097	1	5
GRIP6	women are larger consumers of health care than is actually needed	3.26	1.116	1	5
GRIP7	men do not go to a physician for harmless health problems	3.36	0.954	1	5
GRIP8	medically unexplained symptoms develop in women because they lament too much about their health	3.30	1.021	1	5
GRIP9	female patients complain about their health because they need more attention than male patients	3.60	0.994	2	5
GRIP10	it is easier to find causes of health complaints in men because men communicate in a direct way	3.16	0.965	1	5
GRIP11	men appeal to health care more often with problems they should have prevented	3.37	0.919	2	5
GRIC		2.72	0.660	2.03	3.25
GRIC1	male physicians/nurses put too much emphasis on technical aspects of medicine compared to female physician/nurse	2.51	0.91	1	5
GRIC2	female physicians/nurses extend their consultations too much compared to male physicians/nurses	3.25	0.993	1	5
GRIC3	male physicians/nurses are more efficient than female physicians/nurses	2.03	1.033	1	5
GRIC4 *	female physicians/nurses are more empathic than male physicians/nurses	3.34	1.1	1	5
GRIC5	female physicians needlessly take into account how a patient experiences disease	3.34	0.906	2	5
GRIC6	male physicians/nurses are better able to deal with the work than female physicians/nurses	2.59	1.123	1	5
GRIC7	female physicians/nurses are too emotionally involved with their patients	3.16	1.017	1	5

_R items scored in reverse, i.e., the more you agree, the lower your gender sensitivity score. *: Ex-cluded items; GS: Gender Sensitivity, GRIP: Gender Role Ideology towards patients, GRIC: Gender Role Ideology towards Co-workers.

**Table 3:** Pearson product–moment correlations between measures of gender awareness.

Subscale	1	2	3
1. GS	-		
2. GRI-patient	−0.127	-	
3. GRI-Co-workers	−0.082	0.640 **	-

** Correlation is significant at the 0.01 level (2-tailed).

## Data Availability

The datasets used and analyzed during the current study are available from the corresponding author on reasonable request.
